# Clinical Lectures on Diarrhœa of Infants

**Published:** 1846-11

**Authors:** 


*Clinical Lectures on Diarrhœa of Infants.* By Professor Trousseau.—*Treatment.* We have found few drugs of any avail in the treatment of the bilious diarrhœa in children. It is a convenient plan to call the malady a gastro-enteritis, because the denomination leads to an invariable line of treatment, accessible to understandings of the meanest capacity. We take, however, a different view of diagnosis generally, and deem it unprofitable unless it leads to some practically useful indication. Some forms of diarrhœa are doubtless less difficult of cure than others, but we must say that the varieties we have described often combine with each other, so as to cause the practitioner no small embarrassment, and to reduce him, in many cases, to a blindfold empiricism; not but that we profess much respect for that empiricism which teaches us to exhibit mercury in syphilis, steel in anemia, and bark in ague; but the empiricism we deprecate as a contemptible method is that which is not guided by diagnosis. The method we refer to may become a useful guide to the detection of the nature of disease, and it then acquires a considerable degree of utility. Let us remind you of a case of hemicrania at present in the wards. The attacks were periodical, and we tried sulphate of quinine without success; thus acquiring the knowledge that it was not governed by miasmatic influence. We exhibited then mercurial preparations, and the nervous headache having yielded at once, we were lead to attribute the disease to syphilis. This is the empirical method we adopt; it is not the empiricism of *experiment*, but of *experience*. Thus, if we say that a patient is affected with neuralgia, we express a diagnostic which is as elementary, and, let us add, as useless, as to say that he is affected with a corn on his foot; but it is quite another sort of thing to say the patient is laboring under gouty, syphilitic, miasmatic, rheumatic, or chlorotic neuralgia, because this kind of diagnosis leads us to the real therapeutic indications. To return to the treatment of diarrhœa: Let us not forget that, to arrest the superabundant intestinal secretion is not by any means to cure the complaint which caused it. It is our opinion that bilious diarrhœa is only a very superficial catarrhal derangement of the intestine. The most efficient treatment consists in the exhibition of neutral salts, such as the double tartrate of potass and soda, phosphate of soda, Epsom or Glauber salts. We do not wish you to understand that we recommend the use of purgatives. No; castor oil and magnesia, or manna, you will usually find unsuccessful, whereas the neutral salts generally produce a speedy amendment. We have also derived benefit from the exhibition of pulv. ipecac., at doses varying from two to ten grains, and mixed with a little jam, milk, or simple syrup. The action of this medicine is threefold: it is a substitutive, a discutient, and being a diaphoretic, deviates towards the skin those vital energies which are occupied in the production of those morbid symptoms in the alimentary canal. But when the bilious diarrhœa is the consequence of mere nervous excitement—when it is caused by fear or anger, as tears by grief, or salivation by appetite—opium gives relief in a very short time. In these cases, which you will find to be characterised by the absence of any sort of suffering during the first twenty-four or thirty-six hours, the disease will speedily yield to the influence of hypnotic medicines. Half a drop of Sydenham's laudanum is a sufficient dose for a child under six months; others, it is true, will bear two or three drops, but that dose is too powerful a narcotic for the many. The laudanum should be dissolved in an ounce mixture, whereof the infant shall take a tea-spoonful every three or four hours; but when the disorder has lasted beyond the specified time, opium ceases to possess its salutary effect, because the mere presence of the increased secretions on the mucous surface has sufficed to bring on an irritation which did not exist at first. Then we must again have recourse to neutral salts.

In mucous diarrhœa we have generally derived benefit from three sources: saline purgatives, calomel, and rhubarb. The dose of calomel we recommended is one-fifth of a grain daily, mixed with half a drachm of sugar. This should be continued two or three days at furthest. As to rhubarb, it is the "syrup" we use, the so-called "sirop de chicorée"—a good preparation in everything but its absurd name, which insinuates the idea of the efficacy of the endive, which is, on the contrary, perfectly inert. Great attention should be paid to the child's diet; his food, less abundant than usual, should be chosen with great care. Milk is the most proper food for young children; fecula and broth also may be given after the expiration of the first year, and the drink should be in small quantity. In the choice of food the physician must also allow himself to be guided, in a great measure, by the idiosyncracy of the child, and the mother's remarks on the peculiarities of his appetite.—*Southern Medical and Surgical Journal.—London Med. Times.*

